# Motion sickness in migraine and vestibular disorders

**DOI:** 10.1136/jnnp-2014-308331

**Published:** 2014-08-11

**Authors:** Louisa Murdin, Florence Chamberlain, Sanjay Cheema, Qadeer Arshad, Michael A Gresty, John F Golding, Adolfo Bronstein

**Affiliations:** 1 Department of Neuro-otology, National Hospital for Neurology and Neurosurgery, University College London Hospitals NHS Foundation Trust, London, UK; 2 Guys and St Thomas NHS Foundation Trust, London, UK; 3 Neuro-Otology Unit (Division of Brain Sciences), Imperial College London, Charing Cross Hospital, London, UK; 4 Department of Psychology, University of Westminster, London, UK

**Keywords:** NEUROOTOLOGY

Motion sickness is a syndrome provoked by sensory conflict that involves the vestibular system with symptoms resembling those of common neuro-otological disorders including vestibular neuritis (VN) and vestibular migraine (VM). By contrast, it is generally believed that bilateral vestibular failure (BVF) causes reduced motion sickness susceptibility. We investigate differences between these conditions with a single protocol using validated objective experimental (off-vertical axis rotation, OVAR^1^) and validated patient-centred measures of motion sickness susceptibility.^2^

Five groups were studied:

1. *Normal healthy controls* **(**n=12; mean age 51, SD 17.2; 4/12 women).
2. *VN* (history of acute vertigo without neurological features or hearing loss; none treated with steroids acutely; positive head thrust test; spontaneous unidirectional horizontal nystagmus; acute caloric canal paresis >30%, mean canal paresis repeated in chronic phase after 6 weeks was 38% (SD 31); n=12; disease duration range 10–33 months; mean age 45, SD 15.3; 5/12 women).
3. *BVF* (absent caloric or rotational responses; confirmed in chronic phase; n=8; mean age 51, SD 11.5; 3/8 women).
4. *VM* (recurrent episodic vestibular symptoms in association with migraine according to published criteria with no vestibular test abnormalities^3^; n=12; mean age 45, SD 15.3; 11/12 women).
5. *Migraine without vestibular symptoms* (*M*; recurrent headaches meeting International Headache Society (IHS) 2004 criteria; with/without aura but with no significant vestibular symptoms^3^; n=12; mean age 41, SD 13.6; 8/12 women.

Two groups of patient with migraine were studied (one with vestibular symptoms, VM, and one without vestibular symptoms, M) to determine whether the presence of vestibular symptoms in the setting of migraine influences motion sickness susceptibility. The normal controls and the migraine group were screened for vestibular symptoms but did not undergo formal vestibular testing.

Participants were seated in a motorised chair (Neurokinetics Inc, Pittsburgh, USA). The torso, legs, feet and head were restrained. The chair took 60 s to reach a constant velocity of 72°/s (0.2 Hz) on a vertical axis in the light, then tilted over 20 s to an angle of 18° from earth vertical. Velocity and tilt remained constant until the chair was stopped, when it was brought back to rest and earth vertical over 30 s.

At every minute during rotation, participants rated nausea on a scale from: 1=no symptoms, 2=initial symptoms but no nausea, 3=mild nausea, 4=moderate nausea. Rotation continued until participants Sickness Rating (SR) of 4 or to a maximum of 20 min. To quantify recovery, SRs were also obtained at 1, 2, 3, 4, 5, 10, 15, 20 and 30 min after motion end point.

Individual motion sickness susceptibility was reported before rotation using the Motion Sickness Susceptibility Questionnaire, short form (MSSQ-Short).^2^ Briefly, participants were asked to report retrospectively the frequency of experiencing nausea on various forms of transport/motion. Those in the VN, BVF and VM groups also scored themselves after the onset of vestibular disease.

**Figure 1:**
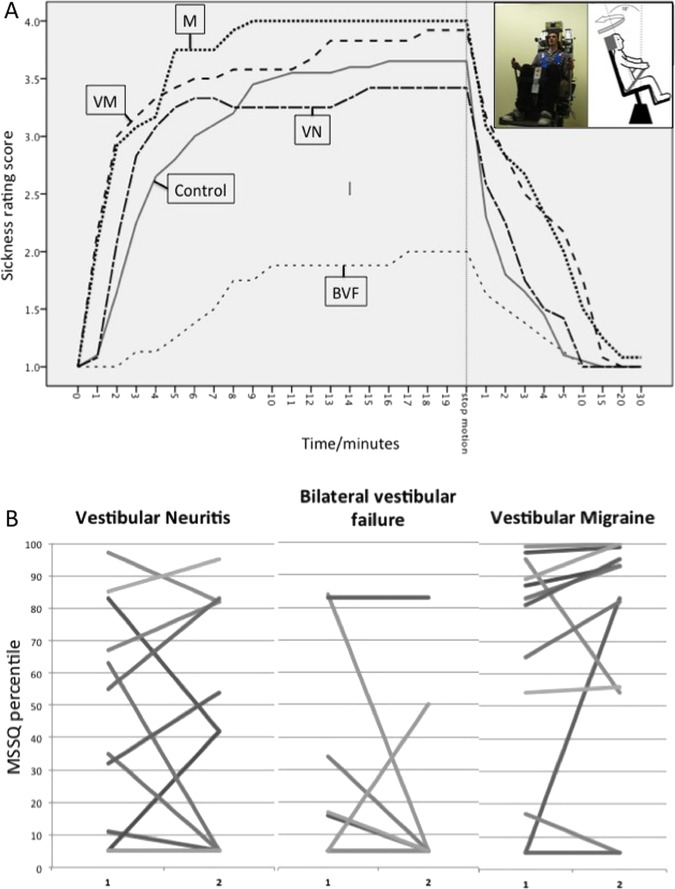
(A) Mean SR (y axis) against time for all five groups (1=no nausea, 2=initial symptoms but no nausea, 3=mild nausea, 4=moderate nausea (stop motion)). In the first frame on the left, time t=0 is at onset of chair motion. For the purposes of illustration, continuation values of Sickness Rating=4 were inserted for those who terminated at moderate nausea before reaching the 20 min motion exposure time cut-off (marked as stop motion on the x axis) From the point of stop motion, time periods on the x axis are denoting length of recovery in minutes (note non-linear scale). The OVAR chair is shown as a small inset. (B) Values for individual participants for MSSQ percentile (y axis) before (x axis point 1) and after (x axis point 2) the onset of vestibular symptoms for VN, BVF and VM groups. The three groups are displayed adjacent and on the same y axis scale for ease of comparison. Note that the majority of individuals in the BVF group experience a reduction in motion sickness susceptibility, while by contrast the majority of the VM group experience an increase in motion sickness susceptibility. The VN group picture is mixed. BVF, bilateral vestibular failure; M, migraine; MSSQ, Motion Sickness Susceptibility Questionnaire; OVAR, off-vertical axis rotation; SR, Sickness Rating; VM, vestibular migraine; VN, vestibular neuritis

Figure 1A shows the mean Sickness Rating scores against time during rotation for all five groups (p<0.001, analysis of variance). Participants who did not reach SR 4 were allocated values of 20 min for analysis. All patients with BVF tolerated motion to 20 min with no scores of SR 4 reported, and 38% patients with BVF remained at SR 1 (no nausea) for the whole test duration with no participant in any other group demonstrating this effect.

Comparing the time to recovery (no nausea) after rotation ceased, there is a significant difference between groups (p<0.001), attributable to the difference between VM/M and the other groups. There was no relationship between side of lesion in the VN group and time to SR 4 (p=0.21, Kruskal-Wallis), and no correlation between MSSQ and canal paresis (p=0.31).

Mean and IQR for MSSQ were (control (10.0, 8.2); VN (13.7, 15.4); BVF (8.5, 5,9); VM (20.7, 13.7) and M (30.1, 15.3) groups; p=0.004). Of the patients with BVF, 13% developed an increased MSSQ score after the onset of their vestibular disorder, and 50% reported decreases. For the VN group, the corresponding result was 42% increased and 42% decreased; for VM 75% increased and 17% decreased. Intraindividual changes are depicted in figure 1B. There was a significant difference between groups (p=0.009) in susceptibility after illness onset, due to the patients with VM and BVF showing oppositely directed trends.

Our study, using validated experimental and questionnaire paradigms, confirms that individuals with BVF report and demonstrate low levels of motion sickness susceptibility that were not present prior to disease acquisition.

Although all BVF individuals could withstand the 20 min rotation, some were not completely immune, perhaps because of residual vestibular function. Some degree of otolith function could still be present since caloric and rotational tests primarily assess horizontal semicircular canal function. It is also known that visual stimuli can provoke symptoms,^4^ and since the experiment took place in the light, this may be an alternative explanation.

Seasickness commonly has a negative impact on leisure and tourism activities such as sailing and cruise travel. Ability to identify positive aspects of a condition can predict outcome in chronic conditions. We therefore recommend that patients with BVF be advised of this beneficial aspect of their condition. Those with VN, a unilateral lesion, do not share this beneficial effect.

There is an overall increase in motion sickness susceptibility of patients with VM but this is not different from migraine. This contrasts with findings of previous studies that have shown higher susceptibility scores in questionnaires in VM than migraine in general.^5^ Unlike any previous study, the questionnaire data from our study are supported by the experimental data. Some individuals with VM reported reductions in susceptibility, suggesting some heterogeneity in the underlying pathomechanism, which could also explain some observed differences between ours and previous studies.

In conclusion, BVF reduces motion sickness susceptibility, and this can be regarded as a beneficial effect of this disorder, but a unilateral lesion is insufficient to trigger such a reduction. VM and migraine similarly enhance motion sickness susceptibility profiles.
